# Effects of CoQ10 Replacement Therapy on the Audiological Characteristics of Pediatric Patients with *COQ6* Variants

**DOI:** 10.1155/2022/5250254

**Published:** 2022-09-09

**Authors:** Dong Woo Nam, Sang Soo Park, So Min Lee, Myung-Whan Suh, Moo Kyun Park, Jae-Jin Song, Byung Yoon Choi, Jun Ho Lee, Seung Ha Oh, Kyung Chul Moon, Yo Han Ahn, Hee Gyung Kang, Hae Il Cheong, Ji Hyun Kim, Sang-Yeon Lee

**Affiliations:** ^1^Department of Otorhinolaryngology, Chungbuk National University Hospital, Cheongju, Republic of Korea; ^2^Department of Otorhinolaryngology-Head and Neck Surgery, Seoul National University Hospital, Seoul National University College of Medicine, Seoul, Republic of Korea; ^3^Department of Otorhinolaryngology, Seoul National University Bundang Hospital, Seongnam, Republic of Korea; ^4^Department of Pathology, Seoul National University Hospital, Seoul, Republic of Korea; ^5^Department of Pediatrics, Seoul National University College of Medicine, Seoul, Republic of Korea; ^6^Department of Pediatrics, Seoul National University Children's Hospital, Seoul, Republic of Korea; ^7^Department of Pediatrics, Hallym University Sacred Heart Hospital, Anyang, Republic of Korea; ^8^Department of Pediatrics, Seoul National University Bundang Hospital, Seongnam, Republic of Korea; ^9^Sensory Organ Research Institute, Seoul National University Medical Research Center, Republic of Korea

## Abstract

Primary coenzyme Q10 (CoQ10) deficiency refers to a group of mitochondrial cytopathies caused by genetic defects in CoQ10 biosynthesis. Primary coenzyme Q10 deficiency-6 (COQ10D6) is an autosomal recessive disorder attributable to biallelic *COQ6* variants; the cardinal phenotypes are steroid-resistant nephrotic syndrome (SRNS), which inevitably progresses to kidney failure, and sensorineural hearing loss (SNHL). Here, we describe the phenotypes and genotypes of 12 children with COQ10D6 from 11 unrelated Korean families and quantitatively explore the beneficial effects of CoQ10 replacement therapy on SNHL. A diagnosis of SRNS generally precedes SNHL documentation. COQ10D6 is associated with progressive SNHL. Four causative *COQ6* variants were identified in either homozygotes or compound heterozygotes: c.189_191delGAA, c.484C>T, c.686A>C, and c.782C>T. The response rate (no further hearing loss or improvement) was 42.9%; CoQ10 replacement therapy may thus limit and even improve hearing loss. Notably, the audiological benefit appeared to be genotype-specific, suggesting a genotype–phenotype correlation. The results of cochlear implantation were generally favorable, and the effects were sustained over time. Our results thus propose the beneficial effects of CoQ10 replacement therapy on hearing loss. Our work with COQ10D6 patients is a good example of personalized, genetically tailored, audiological rehabilitation of patients with syndromic deafness.

## 1. Introduction

Primary coenzyme Q10 (CoQ10) deficiency refers to a group of mitochondrial cytopathies caused by a genetic defect in CoQ10 biosynthesis [1]. Primary CoQ10 deficiency is clinically and genetically heterogeneous. At least 15 genes are involved in biosynthesis, and biallelic mutations in 10 have been reported in association with primary CoQ10 deficiencies in humans: *PDSS1*, *PDSS2*, *COQ2*, *COQ4*, *COQ5*, *COQ6*, *COQ7*, *COQ8A/ADCK3*, *COQ8B/ADCK4*, and *COQ9* [2]. Several clinical phenotypes, including steroid-resistant nephrotic syndrome (SRNS), sensorineural hearing loss (SNHL), encephalopathy, optic atrophy, cardiomyopathy, and weakness, have been reported, depending on the genetic etiology [3, 4]. Early recognition of the causative etiology of primary CoQ10 deficiency is clinically important; exogenous CoQ10 supplementation limits irreversible damage to the kidneys and central nervous system [5], and this is currently the only treatable mitochondrial cytopathy.

Primary coenzyme Q10 deficiency-6 (COQ10D6) is an autosomal recessive disorder attributable to biallelic *COQ6* variants (OMIM # 614650). CoQ10 monooxygenase 6 (COQ6) is a flavin-dependent monooxygenase required for C5-ring hydroxylation during biosynthesis of CoQ10 [2]. COQ6 is highly expressed in the kidney podocytes and the inner ear (stria vascularis and spiral ligament) *in vivo* [4, 6]. Accordingly, the cardinal phenotypes of COQ10D6 are SRNS and SNHL [4, 7]. Histologically, SRNS presents principally as focal segmental glomerulosclerosis (FSGS) inevitably leading to chronic kidney disease (CKD) that is the second-most common cause of kidney failure in the first two decades of life. SRNS of patients with COQ10D6 may respond to CoQ10 replacement therapy that decreases proteinuria and restores normal renal function, particularly if treatment commences before the kidney pathological findings progress [4, 8, 9].

Thus, it has been hypothesized that CoQ10 replacement may prevent or even substantially improve SNHL in patients with COQ10D6. However, in most previous studies, the effects of CoQ10 replacement (or the use of an analog) on SNHL have not been documented in detail. Specifically, no previous study (a total of 30 affected individuals with biallelic *COQ6* variants from 20 families) has quantitatively analyzed the audiological phenotypes [4, 7, 9, 10]; it is thus not possible to scrutinize the audiological spectrum of COQ10D6 or the effects of CoQ10 on hearing loss. A detailed delineation of the audiological phenotypes and the responses to genotype- and mechanism-based drugs would aid our understanding of COQ10D6-related SNHL, allow prediction of the clinical course, shed light on potential genotype–phenotype correlations, and facilitate timely and appropriate audiological rehabilitation.

Here, we describe the phenotypes and genotypes of 12 children from 11 unrelated Korean families with COQ10D6 and quantitatively assess the benefits afforded by exogenous CoQ10 supplement in terms of SNHL. Furthermore, we, for the first time, present the outcomes of cochlear implantation (CI) in those who did not respond to CoQ10 replacement therapy.

## 2. Materials and Methods

### 2.1. Study Participants

This retrospective study was conducted at Seoul National University Children's Hospital, a tertiary center of Korea. From September 2015 to May 2019, 12 pediatric patients (aged <18 years) from 11 unrelated families with biallelic *COQ6* variants were enrolled. Cases lacking hearing test data were excluded. All 12 underwent medical review, auditory phenotyping, and molecular genetic testing. All subjects started taking oral CoQ10 (30 mg/kg daily in three divided doses) after the genetic diagnosis and continued thereafter. The kidney phenotypes and genotypes have been previously reported in 10 patients (except for patients 8-2 and 11) [11, 12]. This study was approved by the Institutional Review Board of Seoul National University Hospital (IRB no. 0905-041-281), which waived the need for informed consent. All methods were performed in accordance with the Declaration of Helsinki (ethical principles for medical research involving human subjects).

### 2.2. Molecular Genetic Testing

In nine patients, *COQ6* variants were detected by Sanger sequencing. The phenotypic combination of steroid-resistant FSGS and SNHL encouraged us to evaluate *COQ6* in these patients [11]. The nucleotide sequence of the primers used in polymerase chain reaction is available upon request. Mutations were annotated using the *COQ6* complementary DNA reference sequence (GenBank Accession no. NM_182476.3). In the remaining three patients, targeted exome sequencing (TES) covering 57 genes known to be associated with SRNS or FSGS at that time was performed, because our strategy for mutational screening of patients with SRNS changed during the course of the study from Sanger sequencing to TES (as a first-tier test). Briefly, genomic DNA was extracted from whole blood using QIAamp DNA mini kits (Qiagen, Valencia, CA, USA). Targeted exome capture was performed using a SureSelect XT customized kit (Agilent Technologies, Santa Clara, CA, USA). Sequencing was performed on the HiSeq 2500 platform (Illumina, San Diego, CA, USA [12]) to generate paired-end 100 bp reads. The mean depths of coverage in sequencing were 310x. Interpretation of variants followed the American College of Medical Genetics and Genomics and the Association for Molecular Pathology (ACMG/AMP) guidelines, and variants classified as pathogenic or likely pathogenic were considered disease-causing mutations [13].

### 2.3. Audiological Profile

We analyzed the results of hearing tests, including auditory brainstem response thresholds (ABRTs) derived using click sounds, auditory steady state responses (ASSRs), play audiometry, and pure tone audiometry (PTA), depending on age. The average hearing threshold was calculated by averaging the air-conduction thresholds at 0.5, 1, 2, and 4 kHz, and the hearing loss was categorized as mild (20–40 dB), moderate (41–55 dB), moderately severe (56–70 dB), severe (71–90 dB), or profound (>90 dB), based on the average hearing thresholds. In cases with residual hearing, only audiograms acquired prior to CI were considered. The effects of CoQ10 replacement therapy on hearing loss were evaluated in 7 patients (14 ears) for whom serial audiograms were available for >1 year after oral CoQ10 administration commenced. For these, we retrieved the hearing thresholds at baseline, during short-term follow-up (within 1 year of initiation of oral CoQ10), and during long-term follow-up (after 1 year). During follow-up, we focused on hearing loss progression after oral CoQ10 administration. As previously described [14], progression was defined when the difference between the lowest and highest mean hearing threshold (i.e., the threshold shift) was >10 dB and the slope of the regression line > 0.5 dB per year. Based on responsiveness to oral CoQ10 supplement therapy, patients with stable hearing or progression in both ears were defined as a responder, whereas a nonresponder with progression of hearing loss in at least one ear. We compared the two groups in terms of sex, age at kidney transplantation, age at commencement of oral administration of CoQ10, mean hearing threshold at that time, age at onset of SRNS and end-stage kidney disease (ESKD), and hearing loss at baseline. We thus sought to exclude any bias imparted by oral CoQ10 efficacy on progression of hearing loss. Hearing thresholds were compared by ear, and the other variables were compared by subject. In addition, the regression gradients during oral CoQ10 supplement therapy were compared by genotype.

### 2.4. Audiological Rehabilitation

For cochlear implantees, speech perception (categorical auditory perception (CAP) scores) was assessed preoperatively and postoperatively at up to five time points. The CAP system has been validated and widely used to assess auditory reception in children [15]; the score ranges from 0 to 7. Speech perception was also evaluated using the Korean version of the Central Institute for the Deaf (K-CID) sentence and Spondee tests and phonetically balanced (PB) word tests, at 70 dB SPL under audio-only conditions at up to five time points during follow-up [16].

### 2.5. Statistical Analyses

All data are shown as means ± standard deviations (SDs). All analyses employed the R statistical package (ver. 3.3.2, R Foundation for Statistical Computing, Vienna, Austria) or Python (ver. 3.8.12, Python Software Foundation, Wilmington, DE, USA). Comparison of sex by group was analyzed using the Fisher exact test, and ages and hearing thresholds were compared using the Wilcoxon rank sum test. Considering limitations such as a small number of patients and heterogeneous duration of follow-up and timing of hearing assessments, the regression gradient for hearing during the treatment period should be considered the point that the data are clustered within one patient [17]. Given this, the clustered Wilcoxon rank sum test using the Datta-Satten method was performed to compare regression gradient during oral CoQ10 supplement therapy, depending on the genotypes. All statistical tests were two-tailed, and *P* < 0.05 was considered significant.

## 3. Results and Discussion

### 3.1. Phenotypes

The phenotypes are listed in Table 1. Of the 12 patients from 11 unrelated families, 6 were male. All presented with SRNS, except patient 11 who presented with nephrotic range proteinuria without nephrotic syndrome, at a median age of 2.7 years (range 0.8–9.1 years). Eight patients progressed to kidney failure at a median of 7.2 (range 1.1–27.0) months after disease onset and underwent kidney transplantation without recurrence of nephropathy in the graft. Kidney biopsy was performed in 11 patients (not in patient 8-2); all exhibited FSGS lesions, including not-otherwise-specified variants in 7, collapsing variants in 3, and a cellular variant in one. Electron microscopy revealed abnormal mitochondrial proliferation (large collections of morphologically abnormal and distorted mitochondria) in the podocytes in 9 patients (Supplementary figure S2, which illustrates the microscopic findings of kidney biopsies of the patients). Patient 10 evidenced no mitochondrial abnormality, and patient 11 was not evaluated because the specimen was inadequate.

Patients 8-1 and 8-2 are siblings. ^∗^Stages of chronic kidney disease (CKD G1–4) are defined as follows: G1: eGFR ≥ 90; G2: eGFR 60–89; G3: eGFR 30–59; G4: eGFR 15–29; ESKD (end-stage kidney disease): eGFR < 15 or treated by dialysis or kidney transplantation (eGFR (estimated glomerular filtration rate) in mL/min/1.73 m^2^ is calculated using the Schwartz formula). ^∗^AKI: acute kidney injury is defined as the abrupt loss of kidney function that results in a decline in GFR: AKI stage (St) 2 and St 3: ≥2.0 and ≥3.0 times increase in serum creatinine from baseline, respectively, within the prior seven days. M: male; F: female; y: years; SRNS: steroid-resistant nephrotic syndrome; PU: proteinuria; FSGS: focal segmental glomerulosclerosis, not-otherwise-specified variant; coll: collapsing variant; cell: cellular variant; CKD: chronic kidney disease; CKD G1–3T: CKD stage 1–3 after transplantation; KT: kidney transplantation; CoQ10: coenzyme Q10; Tx: treatment; SNHL: sensorineural hearing loss; R: right ear; L: left ear; B: both ears; NR: no response; mod-severe: moderately severe; CI: cochlear implant; HA: hearing aids; Mm weak: mild muscle weakness in the lower extremities; DCMP: dilated cardiomyopathy; bilat: bilateral; OA: optic atrophy.

In 10 patients (20 ears), SNHL without a conductive component was detected at the first audiological evaluation (median age 4.7 years, range 0.9–13.9 years) (Supplementary figure S3, which demonstrates the age profile of the *COQ6* variants). The severity of hearing loss at initial evaluation was mild, moderate, moderately severe, severe, and profound in 2 (8.3%), 3 (12.5%), 8 (33.3%), 2 (8.3%), and 5 (20.8%) ears, respectively. All patients except patient 8-1 evidenced symmetrical hearing loss at baseline. In patient 11, hearing remained normal during the follow-up period. A diagnosis of SRNS generally preceded the documentation of SNHL, except for patient 10, with a median interval of 0.5 years (range 0.2–4.1 years). All subjects commenced CoQ10 replacement therapy after genetic diagnosis (at a median of 5.6 years, range 0.9–16 years). Ultimately, 4 patients (7 ears) with bilateral severe to profound SNHL underwent CI.

Ophthalmological abnormalities were found in 3 patients (1 with optic atrophy, 1 with nystagmus, and 1 with both optic atrophy and nystagmus). In addition, mild muscle weakness was detected in 2 patients (16.7%), cardiomyopathy in 1 (8.3%), and atlantoaxial dislocation in 1 (8.3%).

### 3.2. Genotypes

The genotypes are summarized in Table 2. Four different causative *COQ6* variants were identified either homozygous or compound heterozygous: c.189_191delGAA (p.Lys64del), c.484C>T (p.Arg162∗), c.686A>C (p.Gln229Pro), and c.782C>T (p.Pro261Leu). Cosegregation of the variants with the phenotypes of family members, including both parents, was confirmed. All four variants are extremely rare, not only in the Korean Reference Genome Database (KRGDB, 1,722 individuals) (https://coda.nih.go.kr/coda/KRGDB) but also in the Global Minor Allele Frequency (MAF) database that includes the Exome Aggregation Consortium (ExAC) (http://exac.broadinstitute.org/) and genome aggregation (gnomAD) (http://gnomad.broadinstitute.org/) databases. Furthermore, the amino acid residues were highly conserved among the orthologs of several species (Supplementary figure S1, which illustrates the genotypes and domain maps of COQ6 variants), consistent with a high Genomic Evolutionary Rate Profiling (GERP++) score. In addition, in silico analyses predicted that these variants were disease-causing, as evidenced by Combined Annotation Dependent Depletion (CADD) (https://cadd.gs.washington.edu/) and Rare Exome Variant Ensemble Learner (REVEL) (https://sites.google.com/site/revelgenomics/). Accordingly, all four variants satisfy the criteria for pathogenicity of the ACMG/AMP guidelines or the ClinVar standards (Table 2). Our previous haplotype data suggested that both the p.Lys64del and p.Pro261Leu variants shared an ancestral origin in Korea [11].

### 3.3. Effects of CoQ10 Replacement Therapy on Hearing Loss

**Figure 1:**
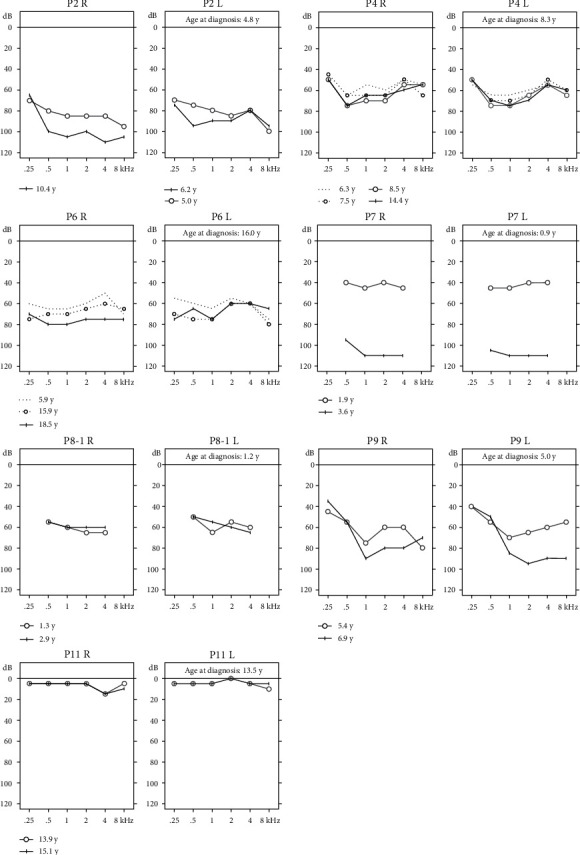
Serial audiograms of 7 patients with biallelic *COQ6* variants. Cases for whom serial audiograms were available for adequate periods after oral CoQ10 administration are shown. Patient 2 underwent left cochlear implantation immediately after the hearing test; the test performed immediately after surgery was excluded from analysis. R: right; L: left; y: years.

**Figure 2:**
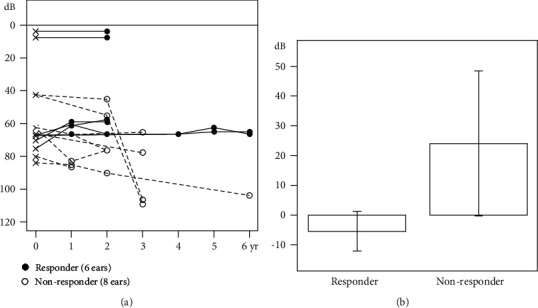
Average hearing thresholds of 7 patients (14 ears) with biallelic *COQ6* variants. (a) Hearing changes by the years of CoQ10 administration. (b) Hearing threshold shifts after CoQ10 administration.

Serial audiograms were available over >1 year after oral CoQ10 administration for 7 patients (14 ears) (Figure 1). The patients were divided into responders (3 patients) and nonresponders (4 patients) using the threshold shift and regression gradient criteria (Figure 2). The mean threshold shift was –5.4 dB (range –18 to 0 dB) in the responder group and 24.1 dB (range –3 to 66 dB) in the nonresponder group (*P* = 0.010, Wilcoxon rank sum test). We found no significant difference in any clinical variable between the two groups (Table 3). In genotype–phenotype correlation analyses, the regression gradient was significantly larger in ears with c.686A>C (*P* = 0.026 by clustered Wilcoxon rank sum test) (Table 4) and significantly smaller in ears with c.189_191delGAA or c.782C>T, but the differences were not statistically significant (*P* = 0.080 and 0.186 in clustered Wilcoxon rank sum test, respectively).

### 3.4. Current Audiological Rehabilitation and Cochlear Implantation Outcomes

**Figure 3:**
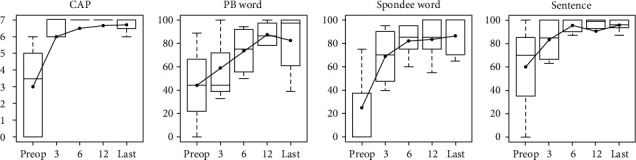
Results of CI in 4 patients (7 ears) with biallelic *COQ6* variants. The CAP and speech perception scores after implantation are shown. All improved rapidly within 1 year after CI, and the improvements were sustained long term to the last follow-up.

In the last audiological evaluation, 11 patients evidenced SNHL with a median hearing threshold of 81.3 dB (range 3.8–118.8 dB). Of these, 7 (63.6%) were using hearing aids and the remaining 4 (36.4%) had undergone unilateral or bilateral CI. In the latter 4 patients (7 ears), the average CAP score was 3.2 (SD: 2.7) at baseline, 6.2 (SD: 1.7) at 3 months postoperatively, 6.5 (SD: 1.2) at 6 months postoperatively, 6.7 (SD: 0.8) at 12 months postoperatively, and 6.7 (SD: 0.5) at the last evaluation (Figure 3). The average follow-up period was 61.9 months (range 24.5–135.5 months). Similarly, the speech perception scores, including the PB and Spondee word scores and K-CID sentence scores, rapidly improved postoperatively (compared to preoperatively) to a level of age-appropriate language development within 1 year.

## 4. Discussion

This is the first study to describe the effects of CoQ10 supplementation on hearing loss in pediatric patients with COQ10D6. Our study is important because we quantitatively analyze the effects of CoQ10 replacement therapy both cross-sectionally and longitudinally in a relatively large cohort of COQ10D6 patients. The proportion of responders was 42.9%, which implies that CoQ10 replacement therapy may thus limit and even improve hearing loss. The audiological benefit appeared to be genotype-specific, suggesting a possible genotype–phenotype correlation. Our work with COQ10D6 patients is a good example of personalized, genetically tailored, audiological rehabilitation of patients with rare syndromic deafness.

### 4.1. Beneficial Effects of CoQ10 Replacement Therapy

There have been very few studies on the beneficial effects of CoQ10 replacement therapy in patients with COQ10D6, and only 10 cases from 7 families in total have been reported [4, 7–10, 18]. Normal hearing was maintained in 2 cases (20%) [8, 18]; hearing loss improved after taking CoQ10 in 1 case (10%) [4] but not in 7 cases (70%) [4, 7, 10]. Rather, 2 cases (20%) newly developed hearing loss after taking CoQ10 (Table 5) [4, 9]. While these previous studies have never been quantitative, the quantitative analyses in our study revealed that 3 of 7 subjects (42.9%) responded positively to CoQ10 supplementation in terms of hearing loss. In detail, normal hearing was maintained in 2 ears (14.3%); 2 ears (14.3%) evidenced hearing improvements ≥ 10 dB; in 4 ears (28.6%), the hearing change ranged from –10 dB to 10 dB; in 6 ears (42.9%), hearing worsened by ≥10 dB. Thus, exogenous CoQ10 supplement may limit and even improve hearing loss in almost half of the patients with COQ10D6.

Mechanistically, *COQ6* is highly expressed in the stria vascularis and spiral ligament of the cochlea [4]. Although the precise mechanism is poorly understood, a severe reduction in COQ6 levels attributable to COQ10D6 may primarily compromise certain steps of endogenous CoQ10 biosynthesis, in turn causing reactive oxygen species- (ROS-) induced cellular damage to the cochlea, and apoptosis, culminating in SNHL. It is conceivable that exogenous CoQ10 supplement replaces endogenous CoQ10 deficiency and ameliorates the vicious circle, maintaining and even improving hearing. Although the cause of individual patient's variability in response to CoQ10 supplementation is not known, it may be explained by individual patient's pharmacokinetics, pharmacodynamics, and tissue trafficking differences [20]. The cochlear blood–labyrinth barrier may hinder CoQ10 delivery since CoQ10 is of high-molecular-weight and low aqueous solubility.

Considering the kidney's perspective on CoQ10 supplementation, 3 (patient 8-2, 9, and 10) of the remaining 6 patients were responder (≥50% reduction in proteinuria, compared to pretreatment state, together with preserved kidney function). Of the 12 patients with COQ10D6, 6 patients (patients 1-6) already in ESKD were excluded from the analysis. Specifically, patient 7 and patient 8-1 did not respond and progressed to ESKD at 7 months and 2 weeks after initiation of CoQ10 therapy, respectively. In patient 8-2 and patient 9, other medications such as calcineurin inhibitor or renin-angiotensin-aldosterone blockades were combined with the CoQ10 supplementation simultaneously, biasing the effect of CoQ10 therapy per se on CoQ6 variants. Additionally, patient 10 received kidney replacement therapy (KRT) for 3 months. As a result, proteinuria was markedly decreased while tapering KRT after CoQ10 treatment. In this study, 4 patients were possible to explore how CoQ10 supplement therapy affects not only hearing but also kidney function simultaneously. Despite lack of cases, it seems that renal function did not correlate with the extent of hearing deterioration (Supplementary table S5, which demonstrates the effect of renal function on the extent of hearing deterioration). Additional evidence awaits further confirmation.

### 4.2. Possible Genotype–Phenotype Correlation on the Effect of CoQ10 Supplementation

It is noteworthy that the effects of CoQ10 supplementation on hearing loss seemed to differ by genotype in this study, suggesting a possible genotype–phenotype correlation. Patients with c.686A>C responded poorly to CoQ10 supplementation, but those with c.189_191delGAA or c.782C>T tended to respond well. Specifically, 1 patient (patient 11) with a homozygous c.782C>T variant exhibited normal hearing at ascertainment, and this did not change over time. Consistent with our data, a previous study reported that SRNS only (thus without SNHL) developed in a COQ10D6 patient homozygous for c.782C>T (Table 5) [18]. It has been tentatively suggested that the genotype–phenotype correlation in certain primary CoQ10 deficiencies correlates with the residual CoQ10 biosynthetic activity evident in yeast [2]. A representative example is *COQ2*; residual enzymatic activity may predict the phenotypic severity [19]. The *COQ6* mutant alleles discussed herein may differ in terms of CoQ10 biosynthetic activity in animal or yeast models, which implies that the audiological benefit afforded by response to CoQ10 supplementation might be genotype-specific. Such a possible genotype–phenotype correlation would aid our understanding of COQ10D6-related SNHL and allow us to better predict the clinical course, facilitating timely and appropriate audiological rehabilitation. Further research is needed.

### 4.3. Polymorphism in *COQ8B*

A previous study that used a yeast model showed that a polymorphism (NM_024876.3, c.521A>G;p.His174Arg) in *COQ8B* affected protein stability and CoQ10 levels [21]. Notably, in siblings with the same *COQ6* variants, *COQ8B* Arg174 alleviated the severity of the renal phenotype compared to that of *COQ8B* His174, suggesting that the *COQ8B* polymorphism may modify the human phenotype [10]. Thus, we analyzed the *COQ8B* polymorphism status of our cohort when possible. Six patients were His174/Arg174 heterozygotes, 3 His174/His174 homozygotes, and 1 an Arg174/Arg174 homozygote (Table 1, Supplementary figure S4, which illustrates the average hearing thresholds before and after administration of CoQ10 to patients with the *COQ8B* SNP). All had SRNS; there were no significant differences in the age of onset (*P* = 0.30, Kruskal-Wallis rank sum test) or the age of onset of hearing loss (*P* = 0.80, Kruskal-Wallis rank sum test) by *COQ8B* polymorphism status. Specifically, in a sibling case, the younger sister (patient 8-2) with *COQ8B* His174/Arg174 exhibited a later age of SRNS onset and maintained normal kidney function to the age of 5.1 years, whereas the older brother (patient 8-1) with *COQ8B* His174/His174 progressed to kidney failure at the age of 1.3 years. In line with this, patient 8-2 evidenced better language development than patient 8-1, although the hearing thresholds were similar. The findings are in line with those of previous studies; *COQ8B* Arg174 may ameliorate the phenotype in terms of the severity of kidney symptoms and the extent of language development.

### 4.4. Results of Cochlear Implantation

Notably, the results of CI were generally favorable in pediatric patients with COQ10D6, as in those with other forms of mitochondrial deafness [22, 23]. Variants in mitochondrial DNA (mtDNA) trigger pleiotropic clinical presentations, including hearing impairment. The auditory phenotype is usually progressive SNHL, ranging from severe to profound deafness to completely normal; most mitochondrial deafness patients evidence bilateral severe to profound deafness, necessitating CI [24]. One systematic review found that CI significantly improved speech perception over time in the absence of significant cognitive deficits [22]. Preoperative identification of the causative variant is crucial from a prognostic perspective because CI requires healthy spiral ganglion neurons (SGNs) [25]. Previous studies have found that most causative variants of mitochondrial deafness localized to presynaptic rather than retrocochlear sites [26], suggesting that CI should be successful. Specifically, in pediatric patients with COQ10D6, auditory performance improved markedly at 1 year postoperatively in terms of both speech perception and sound detection. Further, the effect was sustained long term (average follow-up 50.1 months). Heeringa et al. showed that COQ6 was expressed in multiple cochlear regions, including the stria vascularis, the spiral ligament, and the SGNs of the Rosenthal canal [4]. The traditional classic hypothesis postulated that SGN health predicted CI outcomes [27]. Thus, this would predict that the outcomes of CI in our patients would be poor, in contrast to what we found. Other deviations from the classic hypothesis have also been reported. For example, a recent study described consistently long-term good outcomes after CI in patients with *TMPRSS3* variants [28, 29], despite the fact that TMPRSS3 is expressed in cochlear SGNs [30, 31]. Although SGN degeneration caused by COQ10D6 does trigger a longitudinal decline in performance, timely CI may abate or restore this process, as evidenced herein.

### 4.5. Limitations

This study had certain limitations that should be addressed in the future. In particular, the retrospective nature of the work may limit the generalizability of the results. Although we enrolled one of the largest numbers of pediatric patients with COQ10D6 reported to date, the number remains too small to allow us to draw firm conclusions, including any potential genotype–phenotype correlation. In addition, confounders were not completely assessed; this may have biased the results. Moreover, the lack of a control group (this was not a double-blind, randomized controlled trial) may weaken our findings. As our data is not based upon the case-control study, the results of this study cannot conclude the effect of CoQ10 therapy on COQ6 variants. We therefore suggest that future studies with larger cohorts, together with prospective case-control design, should be directed toward elucidating the effect of CoQ10 therapy on CoQ6 variants. Further studies on families segregating *COQ6* biallelic variants and the establishment of animal models harboring the human genotypes are essential to uncover the audiological characteristics and pathogenic mechanism of hearing loss caused by COQ10D6.

## 5. Conclusions

We have, for the first time, quantitatively analyzed the effects of CoQ10 supplementation on hearing loss in pediatric patients with COQ10D6. Almost half of the patients responded well, proposing the beneficial effects of CoQ10 replacement therapy on hearing loss for cases with COQ10D6. Importantly, the benefit appeared to be specific to the genotype, suggesting a genotype–phenotype correlation. Cochlear implantation significantly improved speech perception over time in patients with COQ10D6. Our work with COQ10D6 patients is a good example of personalized, genetically tailored, audiological rehabilitation of subjects with rare syndromic deafness.

## Figures and Tables

**Table 1:** Phenotypes and genotypes of 12 patients with biallelic *COQ6* variants.

Patients/gender	1/M	2/F	3/F	4/F	5/F	6/M	7/M	8-1/M	8-2/F	9/M	10/M	11/F
Genotypes												
*COQ6*	K64del/P261L	K64del/Q229P	K64del/P261L	K64del/P261L	K64del/P261L	K64del/P261L	Q229P/P261L	K64del/P261L	K64del/P261L	Q229P/P261L	R162∗/P261L	P261L, homozygote
*COQ8B* c.521A>G	A/G	A/G	A/G	A/A	A/A	A/G	A/G	A/A	A/G	G/G	Not done	Not done
Kidney phenotypes												
Age of onset (y)	3.9	2.0	3.9	2.7	1.2	3.1	0.8	1.1	3.0	1.1	9.1	6.9
Mode of onset	SRNS	SRNS	SRNS	SRNS	SRNS	SRNS	SRNS	SRNS	SRNS	SRNS	SRNS	PU
Kidney biopsy	FSGS, coll	FSGS	FSGS, coll	FSGS, coll	FSGS	FSGS	FSGS	FSGS, cell	Not done	FSGS	FSGS	FSGS
Kidney function at start of CoQ10 Tx∗	ESKD	ESKD	ESKD	ESKD	ESKD	ESKD	AKI St 2	CKD G3	Normal	CKD G1	AKI St 3 on CKD G2	CKD G1
Kidney outcome at the last follow-up^∗^	ESKD	ESKD	ESKD	ESKD	ESKD	ESKD	ESKD	ESKD	CKD G1	CKD G1	CKD G3	CKD G2
Age at ESKD (y)	6.2	3.1	4.0	4.6	1.4	3.5	1.5	1.3	—	—	—	—
Age at KT (y)	11.5	6.5	6.3	7.2	6.4	4.9	2.7	2.6	—	—	—	—
Periods from onset to CoQ10 Tx (y)	9.8	2.8	7.1	5.6	13.7	12.9	0.1	0.2	—	3.9	0.9	6.6
Audiological phenotypes												
Age at SNHL (y)	6.0	2.5	4.4	6.3	1.5	5.9	1.2	1.3	5.0	5.2	3.6	—
Age at initial audiometry (y)	12.4	6.0	4.3	2.9	1.6	5.9	0.9	1.2	5.0	5.4	4.2	13.9
Initial hearing level (R/L) (dB)	120/120	84/80	55/60	35/35	NR/NR	60/60	25/25	85/60	59/60	62/62	55/50	7/4
Initial hearing level (R/L) (degree)	Profound	Severe	Moderate/mod-severe	Mild	Profound	Mod-severe	Mild	Severe/mod-severe	Mod-severe	Mod-severe	Moderate	Normal
Threshold difference between two ears (dB)	0	4	5	0	0	0	0	25	1	0	5	3
Symmetry	+	+	+	+	+	+	+	−	+	+	+	+
Rehabilitation status at initial audiometry	(R) CI, (L) HA	(B) HA	None	None	(B) HA	None	None	None	None	None	None	None
Other phenotypes	Mm weak, nystagmus	Exotropia, nystagmus, bilat OA	None	None	Bilat OA	DCMP, Mm weak	None	Atlantoaxial dislocation	None	None	None	None

**Table 2:** *COQ6* variants in the present study and the pathogenicity prediction analyses.

Genomic position (GRCh37/hg19)	HGVS		In silico prediction			Ethnicity MAF	Global MAF	ClinVar/ACMG guideline	
	Nucleotide change	Amino acid change	CADD Phred	REVEL	GERP	KRGDB (1722 individuals)	gnomAD	Classification (criteria)	Reference
14:74420161-74420163	c.189_191delGAA	p.Lys64del	NA	NA	5.37	Absent	0.00001193	Pathogenic	Park et al. [11]
14:74425926	c.782C>T	p.Pro261Leu	33	0.6589	5.55	Absent	0.00006718	Pathogenic	Park et al. [11]
14:74425747	c.686A>C	p.Gln229Pro	27.5	0.698	5.55	Absent	ND	Pathogenic	Park et al. [11]
14:74424852	c.484C>T	p.Arg162∗	38	0.093	4.39	Absent	ND	Pathogenic	Salviati et al. [5] Heeringa et al. [4]

Abbreviations: MAF: minor allele frequency; NA: not available; ND: not detected. Refseq transcript accession number NM_182476.3; Refseq protein accession number NP_872282.1. HGVS: Human Genome Variation Society (https://www.hgvs.org/). Sequence Variant Nomenclature (http://varnomen.hgvs.org/). CADD: Combined Annotation Dependent Depletion (https://cadd.gs.washington.edu/); REVEL: Rare Exome Variant Ensemble Learner (https://sites.google.com/site/revelgenomics/); KRGDB: Korean Reference Genome Database (http://coda.nih.go.kr/coda/KRGDB/index.jsp); gnomAD: Genome Aggregation Database (https://gnomad.broadinstitute.org/). ACMG/AMP 2018 guideline (http://wintervar.wglab.org/).

**Table 3:** A comparison of the clinical variables of responders and nonresponders.

	Responders	Nonresponders	*P* value	Methods
Number of patients	3	4	—	—
Sex (M : F)	1 : 2	3 : 1	0.486	Fisher's exact test
Age at onset of SRNS (years)	3.6 ± 3.0	1.7 ± 1.0	0.629	Wilcoxon rank sum test
Age at onset of ESKD (years)	2.9 ± 2.4	2.7 ± 1.1	1.000	Wilcoxon rank sum test
Age at KT (years)	4.9 ± 3.3	4.7 ± 1.9	1.000	Wilcoxon rank sum test
Age at initiation of CoQ10 administration (years)	7.7 ± 6.2	6.7 ± 6.5	0.857	Wilcoxon rank sum test
Age at onset of SNHL (years)	3.8 ± 3.5	3.7 ± 2.2	0.800	Wilcoxon rank sum test
Average hearing threshold at time point of the initiation of oral CoQ10 administration (dB)^∗^	48.5 ± 33.4	63.4 ± 15.1	0.948	Wilcoxon rank sum test
Threshold shift (dB)^∗^	−5.4 ± 7.3	24.1 ± 26.1	0.010	Wilcoxon rank sum test

^∗^These variables were compared by ears. M: male; F: female; R: right ear; L: left ear; SRNS: steroid-resistant nephrotic syndrome; ESKD: end-stage kidney disease; KT: kidney transplantation; SNHL: sensorineural hearing loss.

**Table:** (a) c.189_191delGAA

	Regression gradient (dB/year)	*P* value/methods
Subjects with c.189_191delGAA in one allele (*n* = 8 ears)	−1.24 ± 6.40	0.080
Subjects without c.189_191delGAA (*n* = 6 ears)	15.4 ± 16.9	Clustered Wilcoxon rank sum test using the Datta-Satten method

**Table:** (b) c.782C>T

	Regression gradient (dB/year)	*P* value/methods
Subjects with c.782C>T in both alleles (*n* = 2 ears)	0.00 ± 0.0	0.186
Subjects with c.782C>T in one allele (*n* = 10 ears)	7.34 ± 16.9	
Subjects without c.782C>T (*n* = 2 ears)	4.42 ± 1.06	Clustered Wilcoxon rank sum test using the Datta-Satten method

**Table:** (c) c.686A>C

	Regression gradient (dB/year)	*P* value/methods
Subjects with c.686A>C in one allele (*n* = 6 ears)	16.8 ± 15.4	0.026
Subjects without c.686A>C (*n* = 8 ears)	−2.35 ± 5.54	Clustered Wilcoxon rank sum test using the Datta-Satten method

**Table 5:** Systematic reviews of the audiological responses to oral CoQ10 administration.

Nucleotide changes	Amino acid substitution	Hearing loss					References
		Maintained normal hearing	Improved	Not improved	Newly developed hearing loss	Total	
c.763G>A, homozygote	p.G255R	0	1	0	1	2	Heeringa et al. [4]
c.782C>T, homozygote	p.P261L	1	0	0	0	1	Gigante et al. [18]
c.1058C>A, homozygote	p.A353D	0	0	1	0	1	Heeringa et al. [4]
		0	0	2	0	2	Perrin et al. [7]
		0	0	2	0	2	Yildirim et al. [19]
c.1078C>T, homozygote	p.R360W	0	0	0	1	1	Cao et al. [9]
c.1078C>T/c.804delC	p.R360W/p.L269Wfs∗13	1	0	0	0	1	Stańczyk et al. [8]
*Total*		**2**	**1**	**5**	**2**	**10**	

## Data Availability

The data that support the findings of this study are available upon request.

## Supplementary Materials

Supplementary 1 Supplementary figure S1: genotypes and domain maps of *COQ6* variants. (a) Exon structures of *COQ6* cDNA and the variants. Variants encountered in our cohort are colored red. (b) Conservation of the affected residues (arrows) among species was documented for all *COQ6* variants observed in the present study.

Supplementary 2 Supplementary figure S2: light microscopic (a) and electron microscopic (b) findings of kidney biopsies of the patients with biallelic *COQ6* mutations manifesting as steroid-resistant focal segmental glomerulosclerosis and sensorineural hearing loss. A glomerulus featuring segmental sclerosis of the not-otherwise-specified variant (a: patient 9, ×400, periodic acid-Schiff staining). A large collection of morphologically abnormal and distorted mitochondria (arrows) in podocyte cytoplasm (b: patient 8-1).

Supplementary 3 Supplementary figure S3: age profile of the *COQ6* variants. (a) The age of onset of disease and the clinical courses are indicated. (b) The ages at SRNS (red circles), SNHL (white circles), and genetic diagnosis (green circles) are illustrated for each patient. The age at genetic diagnosis became lower over time.

Supplementary 4 Supplementary figure S4: the average hearing thresholds before and after administration of CoQ10 to patients with the *COQ8B* SNP. The average hearing thresholds were divided into three groups by the *COQ8B* c.521A>G SNP: A/A homozygotes, A/G heterozygotes, and G/G homozygotes. The horizontal axis is the average hearing threshold before administration, and the vertical axis is that after administration.

Supplementary 5 Supplementary table S5: effect of renal function on the extent of hearing deterioration.

## References

[1] Quinzii C. M., Hirano M. (2011). Primary and secondary CoQ(10) deficiencies in humans. *BioFactors*.

[2] Alcázar-Fabra M., Rodríguez-Sánchez F., Trevisson E., Brea-Calvo G. (2021). Primary coenzyme Q deficiencies: a literature review and online platform of clinical features to uncover genotype-phenotype correlations. *Free Radical Biology and Medicine*.

[3] Scalais E., Chafai R., Van Coster R. (2013). Early myoclonic epilepsy, hypertrophic cardiomyopathy and subsequently a nephrotic syndrome in a patient with CoQ10 deficiency caused by mutations in para-hydroxybenzoate-polyprenyl transferase (COQ2). *European Journal of Paediatric Neurology*.

[4] Heeringa S. F., Chernin G., Chaki M. (2011). COQ6 mutations in human patients produce nephrotic syndrome with sensorineural deafness. *The Journal of Clinical Investigation*.

[5] Salviati L., Trevisson E., Doimo M., Navas P. (2017). *Primary Coenzyme Q10 Deficiency*.

[6] Ozeir M., Mühlenhoff U., Webert H., Lill R., Fontecave M., Pierrel F. (2011). Coenzyme Q biosynthesis: Coq6 is required for the C5-hydroxylation reaction and substrate analogs rescue Coq6 deficiency. *Chemistry & Biology*.

[7] Justine Perrin R., Rousset-Rouvière C., Garaix F. (2020). COQ6mutation in patients with nephrotic syndrome, sensorineural deafness, and optic atrophy. *JIMD Reports*.

[8] Stańczyk M., Bałasz-Chmielewska I., Lipska-Ziętkiewicz B., Tkaczyk M. (2018). CoQ10-related sustained remission of proteinuria in a child with COQ6 glomerulopathy-a case report. *Pediatric Nephrology*.

[9] Cao Q., Li G. M., Xu H. (2017). Coenzyme Q(10) treatment for one child with COQ6 gene mutation induced nephrotic syndrome and literature review. *Chinese Journal of Pediatrics*.

[10] Yuruk Yildirim Z., Toksoy G., Uyguner O. (2020). Primary coenzyme Q10 deficiency-6 (COQ10D6): two siblings with variable expressivity of the renal phenotype. *The European Journal of Medical Genetics*.

[11] Park E., Ahn Y. H., Kang H. G. (2017). COQ6 mutations in children with steroid-resistant focal segmental glomerulosclerosis and sensorineural hearing loss. *American Journal of Kidney Diseases*.

[12] Park E., Lee C., Kim N. K. D. (2020). Genetic study in Korean pediatric patients with steroid-resistant nephrotic syndrome or focal segmental glomerulosclerosis. *Journal of Clinical Medicine*.

[13] Richards S., Aziz N., Bale S. (2015). Standards and guidelines for the interpretation of sequence variants: a joint consensus recommendation of the American College of Medical Genetics and Genomics and the Association for Molecular Pathology. *Genetics in Medicine*.

[14] Colvin I. B., Beale T., Harrop-Griffiths K. (2006). Long-term follow-up of hearing loss in children and young adults with enlarged vestibular aqueducts: relationship to radiologic findings and Pendred syndrome diagnosis. *The Laryngoscope*.

[15] Archbold S., Lutman M. E., Marshall D. H. (1995). Categories of auditory performance. *The Annals of otology, rhinology & laryngology*.

[16] Lee S.-Y., Choi B. Y. (2021). Potential implications of slim modiolar electrodes for severely malformed cochleae: a comparison with the straight array with circumferential electrodes. *Clinical and experimental otorhinolaryngology*.

[17] Datta S., Satten G. A. (2005). Rank-sum tests for clustered data. *Journal of the American Statistical Association*.

[18] Gigante M., Diella S., Santangelo L. (2017). Further phenotypic heterogeneity of CoQ10 deficiency associated with steroid resistant nephrotic syndrome and novel COQ2 and COQ6 variants. *Clinical Genetics*.

[19] Desbats M. A., Morbidoni V., Silic-Benussi M. (2016). The COQ2 genotype predicts the severity of coenzyme Q10 deficiency. *Human Molecular Genetics*.

[20] Horvath R. (2012). Update on clinical aspects and treatment of selected vitamin-responsive disorders II (riboflavin and CoQ 10). *The Journal of Inherited Metabolic Disease*.

[21] Vazquez Fonseca L., Doimo M., Calderan C. (2018). Mutations in COQ8B (ADCK4) found in patients with steroid-resistant nephrotic syndrome alter COQ8B function. *Human Mutation*.

[22] Zia N., Nikookam Y., Muzaffar J., Kullar P., Monksfield P., Bance M. (2021). Cochlear implantation outcomes in patients with mitochondrial hearing loss: a systematic review and narrative synthesis. *The Journal of International Advanced Otology*.

[23] Yamamoto N., Okuyama H., Hiraumi H., Sakamoto T., Matsuura H., Ito J. (2015). The outcome of cochlear implantation for mitochondrial disease patients with syndromic hearing loss. *Otology & Neurotology*.

[24] Yelverton J. C., Arnos K., Xia X.-J., Nance W. E., Pandya A., Dodson K. M. (2013). The clinical and audiologic features of hearing loss due to mitochondrial mutations. *Otolaryngology–Head and Neck Surgery*.

[25] Lee S.-Y., Shim Y. J., Han J.-H. (2020). The molecular etiology of deafness and auditory performance in the postlingually deafened cochlear implantees. *Scientific Reports*.

[26] Vandana V. P., Bindu P. S., Sonam K. (2016). Audiological manifestations in mitochondrial encephalomyopathy lactic acidosis and stroke like episodes (MELAS) syndrome. *Clinical Neurology and Neurosurgery*.

[27] Shearer A. E., Eppsteiner R. W., Frees K. (2017). Genetic variants in the peripheral auditory system significantly affect adult cochlear implant performance. *Hearing Research*.

[28] Moon I. S., Grant A. R., Sagi V., Rehm H. L., Stankovic K. M. (2021). TMPRSS3 gene variants with implications for auditory treatment and counseling. *Frontiers in Genetics*.

[29] Holder J. T., Morrel W., Rivas A., Labadie R. F., Gifford R. H. (2021). Cochlear implantation and electric acoustic stimulation in children with TMPRSS3 genetic mutation. *Otology & Neurotology*.

[30] Guipponi M., Vuagniaux G., Wattenhofer M. (2002). The transmembrane serine protease (TMPRSS3) mutated in deafness DFNB8/10 activates the epithelial sodium channel (ENaC) in vitro. *Human Molecular Genetics*.

[31] Guipponi M., Toh M.-Y., Tan J. (2008). An integrated genetic and functional analysis of the role of type II transmembrane serine proteases (TMPRSSs) in hearing loss. *Human Mutation*.

