# Supplementary material for: Effects of CoQ10 Replacement Therapy on the Audiological Characteristics of Pediatric Patients with COQ6 Variants
Source: Biomed Res Int. 2022 Sep 9;2022:5250254. doi: 10.1155/2022/5250254 (PMC9482153; doi:10.1155/2022/5250254)
Supplement: Supplementary 1 — Supplementary figure S1: genotypes and domain maps of COQ6 variants. (a) Exon structures of COQ6 cDNA and the variants. Variants encountered in our cohort are colored red. (b) Conservation of the affected residues (arrows) among species was documented for all COQ6 variants observed in the present study. [file 5250254.f1.pdf]

Effects of CoQ10 replacement therapy on the audiological characteristics of pediatric patients with *COQ6* variants

Dong Woo Nam, Sang Soo Park, So Min Lee, Myung-Whan Suh, Moo Kyun Park, Jae-Jin Song, Byung Yoon Choi, Jun Ho Lee, Seung Ha Oh, Kyung Chul Moon, Yo Han Ahn, Hee Gyung Kang, Hae Il Cheong, Ji Hyun Kim, Sang-Yeon Lee

Fig.S1

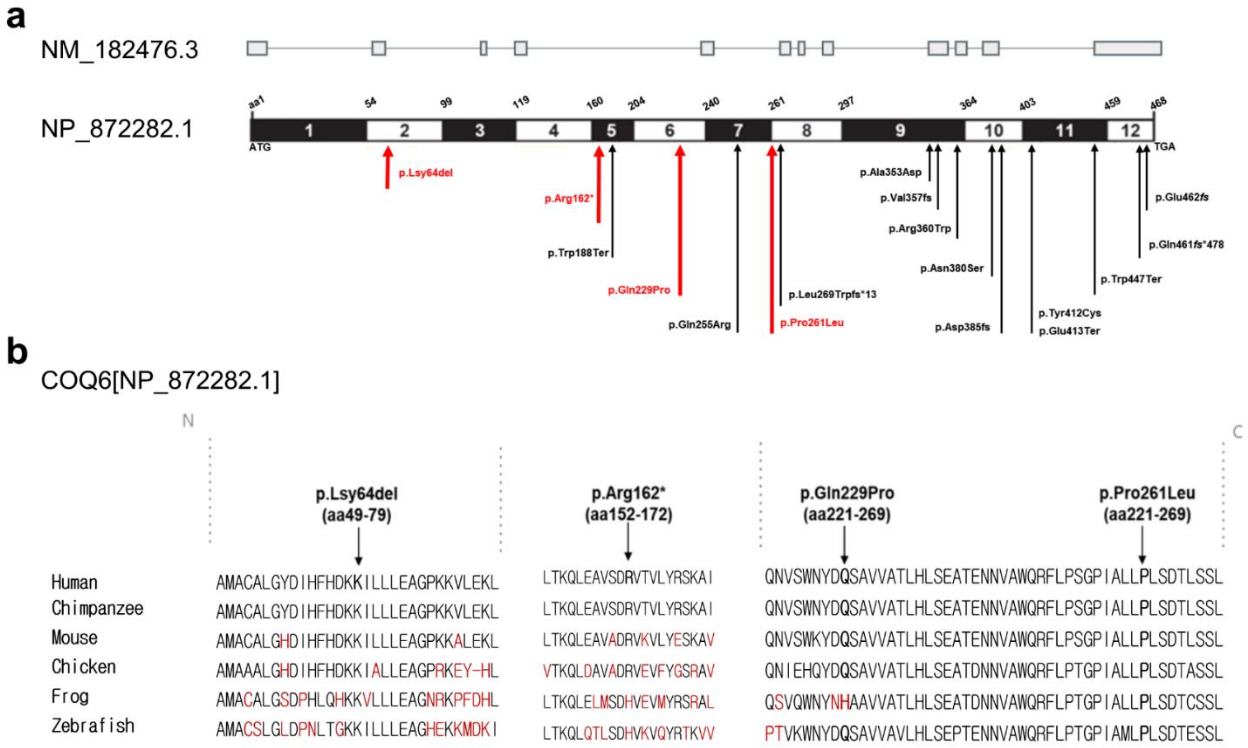

Supplementary figure S1. Genotypes and domain maps of *COQ6* variants. (a) Exon structures of *COQ6* cDNA and the variants. Variants encountered in our cohort are colored red. (b) Conservation of the affected residues (arrows) among species was documented for all *COQ6* variants observed in the present study.
