# Supplementary material for: Effects of CoQ10 Replacement Therapy on the Audiological Characteristics of Pediatric Patients with COQ6 Variants
Source: Biomed Res Int. 2022 Sep 9;2022:5250254. doi: 10.1155/2022/5250254 (PMC9482153; doi:10.1155/2022/5250254)
Supplement: Supplementary 2 — Supplementary figure S2: light microscopic (a) and electron microscopic (b) findings of kidney biopsies of the patients with biallelic COQ6 mutations manifesting as steroid-resistant focal segmental glomerulosclerosis and sensorineural hearing loss. A glomerulus featuring segmental sclerosis of the not-otherwise-specified variant (a: patient 9, ×400, periodic acid-Schiff staining). A large collection of morphologically abnormal and distorted mitochondria (arrows) in podocyte cytoplasm (b: patient 8-1). [file 5250254.f2.pdf]

Effects of CoQ10 replacement therapy on the audiological characteristics of pediatric patients with *COQ6* variants

Dong Woo Nam, Sang Soo Park, So Min Lee, Myung-Whan Suh, Moo Kyun Park, Jae-Jin Song, Byung Yoon Choi, Jun Ho Lee, Seung Ha Oh, Kyung Chul Moon, Yo Han Ahn, Hee Gyung Kang, Hae Il Cheong, Ji Hyun Kim, Sang-Yeon Lee

Fig.S2

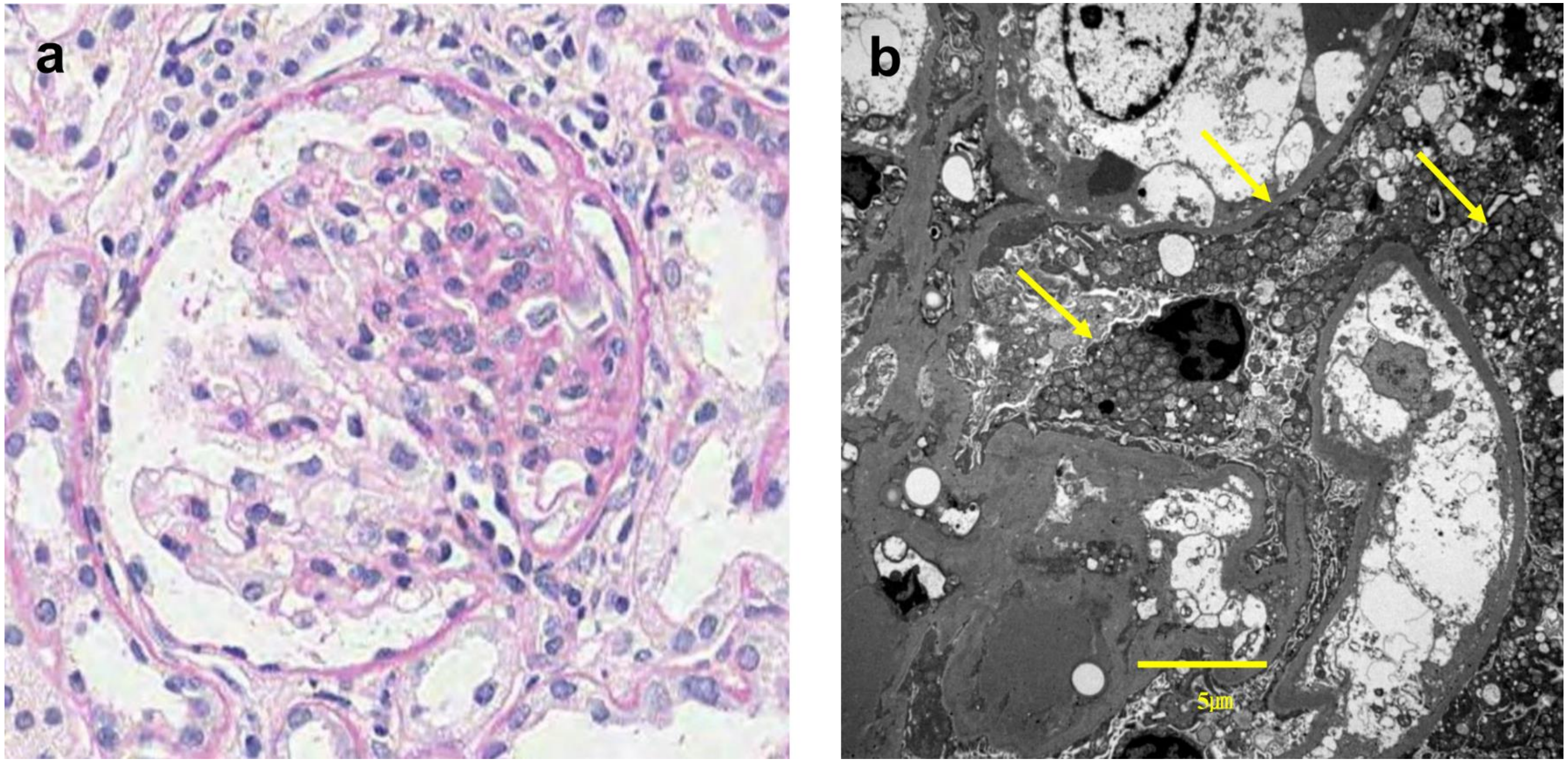

Supplementary figure S2. Light microscopic (a) and electron microscopic (b) findings of kidney biopsies of the patients with biallelic *COQ6* mutations manifesting as steroid-resistant focal segmental glomerulosclerosis and sensorineural hearing loss. A glomerulus featuring segmental sclerosis of the not-otherwise-specified variant (a: Patient 9, 400×, periodic acid-Schiff staining). A large collection of morphologically abnormal and distorted mitochondria (arrows) in podocyte cytoplasm (b: Patient 8-1).
