# Supplementary material for: Effects of CoQ10 Replacement Therapy on the Audiological Characteristics of Pediatric Patients with COQ6 Variants
Source: Biomed Res Int. 2022 Sep 9;2022:5250254. doi: 10.1155/2022/5250254 (PMC9482153; doi:10.1155/2022/5250254)
Supplement: Supplementary 3 — Supplementary figure S3: age profile of the COQ6 variants. (a) The age of onset of disease and the clinical courses are indicated. (b) The ages at SRNS (red circles), SNHL (white circles), and genetic diagnosis (green circles) are illustrated for each patient. The age at genetic diagnosis became lower over time. [file 5250254.f3.pdf]

Effects of CoQ10 replacement therapy on the audiological characteristics of pediatric patients with *COQ6* variants

Dong Woo Nam, Sang Soo Park, So Min Lee, Myung-Whan Suh, Moo Kyun Park, Jae-Jin Song, Byung Yoon Choi, Jun Ho Lee, Seung Ha Oh, Kyung Chul Moon, Yo Han Ahn, Hee Gyung Kang, Hae Il Cheong, Ji Hyun Kim, Sang-Yeon Lee

Fig.S3

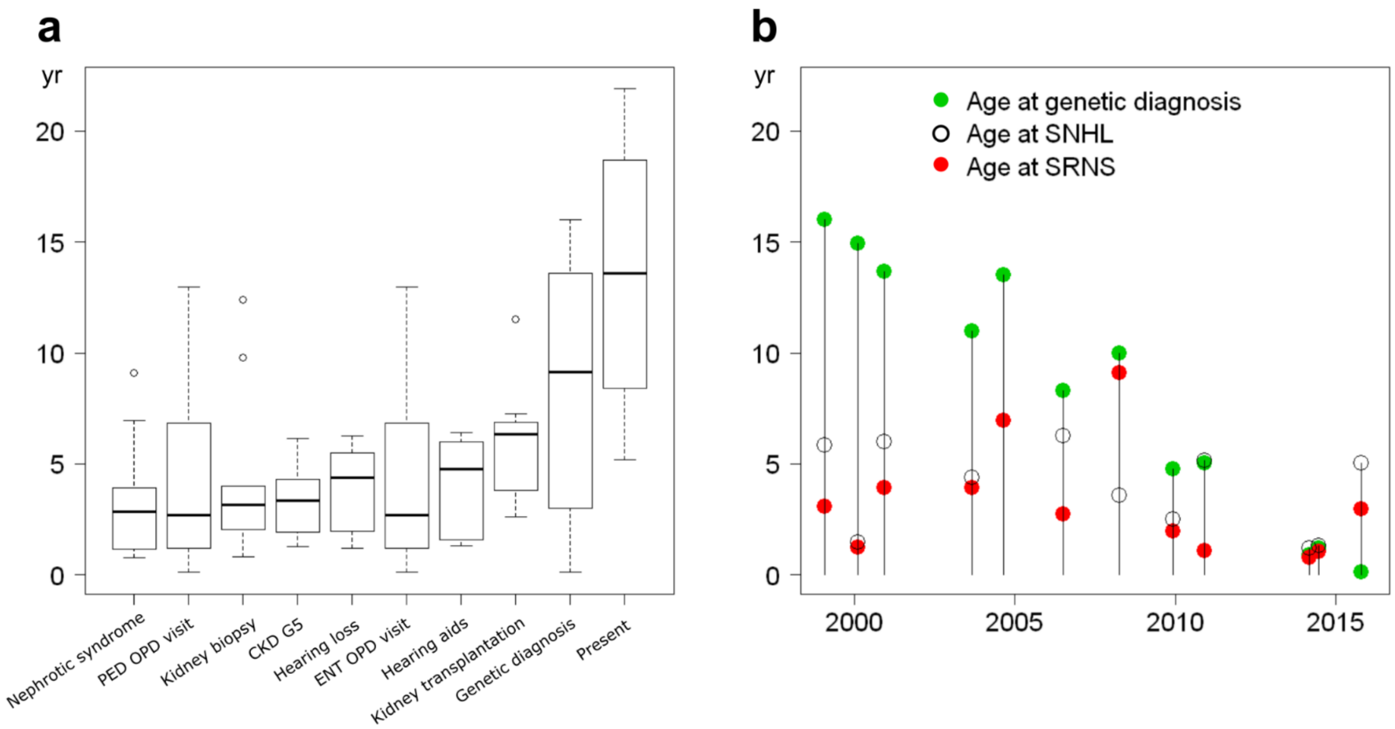

Supplementary figure S3. Age profile of the *COQ6* variants. (a) The age of onset of disease and the clinical courses are indicated. (b) The ages at SRNS (red circles), SNHL (white circles), and genetic diagnosis (green circles) are illustrated for each patient. The age at genetic diagnosis became lower over time.
