# Supplementary material for: Effects of CoQ10 Replacement Therapy on the Audiological Characteristics of Pediatric Patients with COQ6 Variants
Source: Biomed Res Int. 2022 Sep 9;2022:5250254. doi: 10.1155/2022/5250254 (PMC9482153; doi:10.1155/2022/5250254)
Supplement: Supplementary 4 — Supplementary figure S4: the average hearing thresholds before and after administration of CoQ10 to patients with the COQ8B SNP. The average hearing thresholds were divided into three groups by the COQ8B c.521A>G SNP: A/A homozygotes, A/G heterozygotes, and G/G homozygotes. The horizontal axis is the average hearing threshold before administration, and the vertical axis is that after administration. [file 5250254.f4.pdf]

Effects of CoQ10 replacement therapy on the audiological characteristics of pediatric patients with *COQ6* variants

Dong Woo Nam, Sang Soo Park, So Min Lee, Myung-Whan Suh, Moo Kyun Park, Jae-Jin Song, Byung Yoon Choi, Jun Ho Lee, Seung Ha Oh, Kyung Chul Moon, Yo Han Ahn, Hee Gyung Kang, Hae Il Cheong, Ji Hyun Kim, Sang-Yeon Lee

Fig.S4

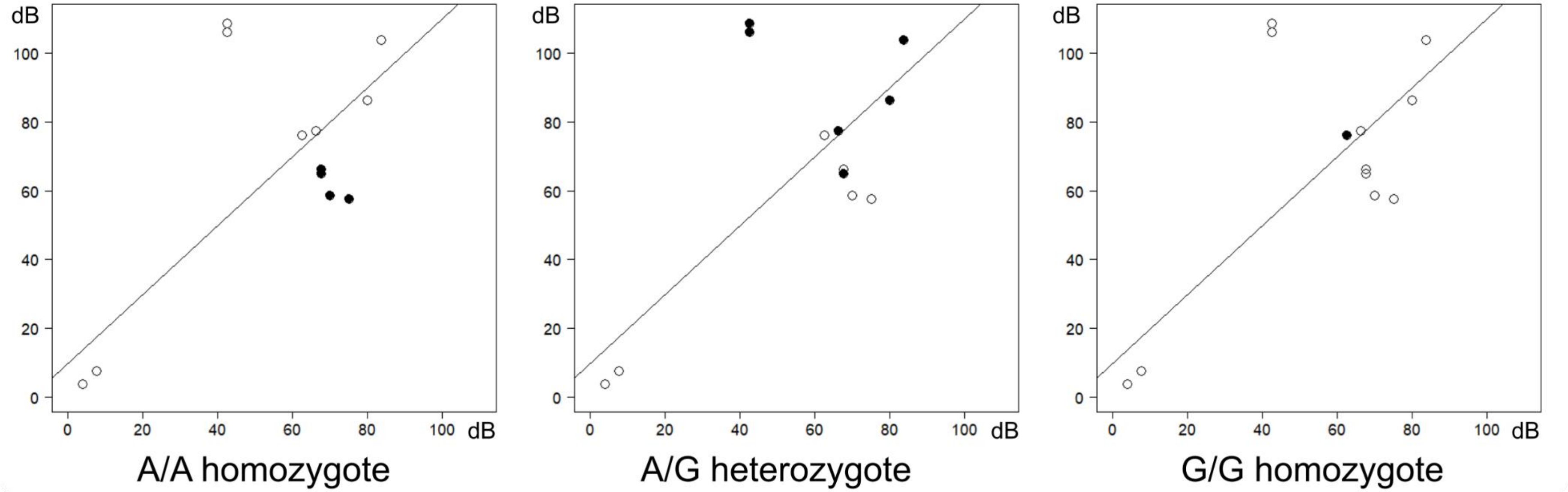

Supplementary figure S4. The average hearing thresholds before and after administration of CoQ10 to patients with the *COQ8B* SNP. The average hearing thresholds were divided into three groups by the *COQ8B* c.521A>G SNP: A/A homozygotes, A/G heterozygotes, and G/G homozygotes. The horizontal axis is the average hearing threshold before administration, and the vertical axis that after administration.
