# Supplementary material for: Effects of CoQ10 Replacement Therapy on the Audiological Characteristics of Pediatric Patients with COQ6 Variants
Source: Biomed Res Int. 2022 Sep 9;2022:5250254. doi: 10.1155/2022/5250254 (PMC9482153; doi:10.1155/2022/5250254)
Supplement: Supplementary 5 — Supplementary table S5: effect of renal function on the extent of hearing deterioration. [file 5250254.f5.pdf]

**Effects of CoQ10 replacement therapy on the audiological characteristics of pediatric patients with *COQ6* variants**

Dong Woo Nam, Sang Soo Park, So Min Lee, Myung-Whan Suh, Moo Kyun Park, Jae-Jin Song, Byung Yoon Choi, Jun Ho Lee, Seung Ha Oh, Kyung Chul Moon, Yo Han Ahn, Hee Gyung Kang, Hae Il Cheong, Ji Hyun Kim, Sang-Yeon Lee

# Table.S5

**Supplementary table S5**. Effect of renal function on the extent of hearing deterioration

| | Patient 7 | Patient 8-1 | Patient 9 | Patient 11 |
|---|---|---|---|---|
| *COQ6* Genotype | Q229P/ P261L | K64del/ P261L | Q229P/ P261L | P261L/ P261L |
| Change of renal function after CoQ10 administration | Aggravated | Aggravated | Improved | Not changed |
| Classification of hearing after CoQ10 administration | Non-responder | Responder | Non-responder | Responder |
| Correlation of renal function with extent of hearing deterioration | Possible | No | No | No |

Note: Four patients were possible to analyze how CoQ10 supplement therapy affects not only hearing but also kidney function simultaneously. As for Patient 1-6, the renal function was in progress to ESKD before CoQ10 administration. As for Patient 8-2 and Patient 10, audiological follow-up was not available for more than 1 year after CoQ10 administration, thereby excluding from this analysis.
